# The Relationships of Creative Coping and College Students’ Achievement Emotions and Academic Stress: The Mediating Role of Psychological Capital

**DOI:** 10.3390/jintelligence10040126

**Published:** 2022-12-15

**Authors:** Chenxin Xu, Qing Wang

**Affiliations:** Shanghai Key Laboratory of Mental Health and Psychological Crisis Intervention, School of Psychology and Cognitive Science, East China Normal University, Shanghai 200062, China

**Keywords:** academic stress, achievement emotions, creativity, creative coping, psychological capital

## Abstract

Creative coping is the use of creativity as a positive strategy when facing stress. The existing empirical investigation of creative coping is scarce, particularly in the field of educational psychology. The present study aims to explore the relationships of college students’ creative coping and their achievement emotions and academic stress as well as the underlying mechanism. The sample included 780 Chinese college students. The Creative Coping Scale, Positive Psychological Capital Questionnaire, Learning Stress Inventory for College Students, and the short version of the Achievement Emotions Questionnaire were used. Statistical results showed that creative coping was positively related with students’ positive achievement emotions and negatively related with negative achievement emotions, but insignificantly with academic stress. Moreover, psychological capital played a mediating role in the relationship between creative coping and achievement emotions and in the relationship between creative coping and academic stress with a suppression effect.

## 1. Introduction

Students encounter stress in their daily academic life that is often accompanied by negative achievement emotions (Alzahrani et al. 2020; Arsenio and Loria 2014; Neveu et al. 2012; Pekrun 2006; Zhang and Zheng 2017). Therefore, students need to regulate their emotions with coping strategies, such as problem-focused and emotion-focused coping (Folkman and Lazarus 1980), or more specific ones such as planning, active coping, restraint, acceptance, and so on (Carver et al. 1989). According to Lazarus and Folkman (1984), coping is the process of executing potential responses to the threat, including appraising the level of threat, choosing a response, and reappraising whether it is appropriate. Most of the time, people use common or habitual ways to cope with stress, for instance strategizing, wishful thinking, support-seeking, and so on (Salimzadeh et al. 2021). However, people may tend to manage demands regardless of the success of the efforts (Folkman 1984). Creative coping is the use of creativity as a positive coping strategy when facing stress (De Fazio et al. 2019). It indicates that people actively think about new approaches to coping and deliberately try something never used before. For example, writing a poem or taking a photo when feeling blue. When peopleexercise their creativity in coping, they may expand their cognitive repertoire, discover more personal resources, and come up with novel and effective ways to deal with demanding situations. The effect of creative coping on emotion regulation and stress reduction is yet unknown in education literature. The present study aims to explore the associations of creative coping and college students’ achievement emotions and academic stress, as well as the underlying mechanisms.

### 1.1. The Relationships of Creative Coping, Academic Stress and Achievement Emotions

College students commonly experience academic stress: solving academic problems, managing their time, encountering loneliness and burnout, and facing other academic stressors (Kausar 2010; Stoliker and Lafreniere 2015). Academic stress is strongly related to achievement emotions that are linked with students’ learning, including positive emotions (e.g., enjoyment, hope, pride) and negative ones (e.g., anger, anxiety, hopelessness, boredom) (Pekrun et al. 2011, 2017). Previous studies have found that higher academic stress is related to students’ overall moods; academic stress is positively related to negative academic affect and negatively related to positive emotions (Arsenio and Loria 2014; Santiago et al. 2017). To be specific, Wang et al. (2019) found that students’ perceived stress was negatively correlated with pride and relief; Stupnisky et al. (2013) found that perceived stress was positively correlated with anxiety and boredom.

There are abundant studies on how to cope with academic stress and regulate achievement emotions (Cherry and Wilcox 2020; Harley et al. 2019; Mirsamiei et al. 2021; Yousif et al. 2022). The coping methods for academic stress can be generally categorized into positive or negative methods (Cherkil et al. 2013; Zhou et al. 2017). For example, task-focused coping is regarded as a positive coping method for test-related academic anxiety (Putwain et al. 2016), whereas avoidance coping and social-focused coping are not as effective as task-focused methods in dealing with specific academic problems (Thomas et al. 2017). Moreover, acceptance, positive reframing, using humor, planning, and active coping are positive coping methods for academic stress (Darabi et al. 2017). In terms of how to regulate achievement emotions, previous studies found that strategies such as acceptance of emotions, deep breathing, and positive self-expression would help students to regulate their negative achievement emotions and enhance academic quality (Mirsamiei et al. 2021). Reappraisal strategies are generally more effective and adaptive than suppression strategies (Leroy et al. 2012; Strain and D’Mello 2015) and positively correlated with students’ self-efficacy beliefs (Zyberaj 2022).

Creativity is one potential mechanism that has been investigated to facilitate stress coping (Norma Contini de 2016) and dealing with students’ achievement emotions or depression (Alfonso-Benlliure and Melendez 2022; Ding et al. 2014). Creative coping is the use of creativity as a positive coping strategy when facing stress (De Fazio et al. 2019). Based on the theory of transformative coping (Corry et al. 2013, 2014), creativity, with its transformative quality and association with spirituality, can lead to a reduction in negative emotions (De Fazio et al. 2019; Kimport and Robbins 2012) as well as the transformation of negative emotions into positive ones and more effective long-term coping (Corry et al. 2014). Creativity can influence how one adapts to college experience and how one acquires coping skills for different stressors (De Fazio et al. 2019). Students may optimize their creativity resource to cope with stress. For instance, Falat (2000) found that highly creative students would utilize significantly more active strategies in coping with frustrating situations. Students who reappraise stressor as a challenge to personal growth and accomplishment obtained higher scores in creativity (Li et al. 2018). In addition, Park and Kim (2018) found that Korean adolescents created their own coping strategies to deal with various academic stressors, which can be regarded as the application of creativity in coping with academic pressure. Although many studies showed that creativity is conducive to coping, there is evidence that may support a counter-hypothesis when it comes to the relationship between creative coping and academic stress. For example, Khan et al. (2014) found no significant correlation between problem solving coping style and academic stress, and An et al. (2012) found that students who are novelty-seeking, which is linked to creativity (Gocłowska et al. 2019), tend to have a higher level of stress. However, the evidence above indicated a general coping style or creativity as the independent variable; indeed, there is no empirical study directly investigating into the relationship between creative coping and academic stress. Moreover, people use coping strategies in order to reduce their stress, but the effects can be diverse in reality—people may or may not achieve their aims. Therefore, we tentatively hypothesized that creative coping is negatively associated with academic stress. On the other hand, empirical evidence has supported the relationship between creativity and emotion regulation. Yeh and Li (2008) found that preschool children’s emotion regulation strategies had a strong predictive power of creativity (*β* = .82). Pavlova and Kornilova (2013) found that creativity, emotional intelligence, and tolerance of uncertainty acted as predictors of the use of emotional information in decision making. Moreover, a diary study showed that people who reported more creative activities than usual felt higher activated positive affect and flourishing in the following days (Conner et al. 2018), showing that engaging in creative behavior can lead to an increase of positive emotions.

Based on the aforementioned literature, we propose the following hypotheses:

**Hypothesis** **1.** *Creative coping is positively associated with positive achievement emotions and negatively associated with academic stress and negative achievement emotions in college students.*

**Hypothesis** **2.** *Creative coping can positively predict positive achievement emotions and negatively predict academic stress and negative achievement emotions in college students.*

### 1.2. The Potential Mediating Role of Psychological Capital

Psychological capital (PsyCap) refers to an individual’s positive psychological resource that includes four essential psychological capacities: (1) self-efficacy, the belief that one can accomplish a challenging task successfully; (2) hope, perseverance toward goals and redirection of paths to accomplish goals when necessary; (3) optimism, positive expectations about present and future success; and (4) resilience, the ability to bounce back and even beyond to attain success when beset by problems and adversity (Luthans et al. 2004, 2006, 2007). Creativity is found to be positively related to PsyCap (Cai et al. 2019; Huang and Luthans 2015; Liu et al. 2020). Specifically, in the context of learning, creativity can be positively related to self-efficacy (Wu et al. 2017), optimism, and hope (Zhang et al. 2019), and it can be a positive predictor of resilience (Chen and Padilla 2019). Positive coping strategies are found to have a positive relationship with PsyCap (e.g., Rabenu et al. 2016). Various positive coping methods positively relate to hope, optimism, and self-efficacy (Darabi et al. 2017), foster hope (Folkman 2010), and predict resilience (Denovan and Macaskill 2017). As one kind of positive coping strategy, creative coping and PsyCap may be positively correlated. When students use a creative way to cope with academic stress and regulate emotions, they may gain experience of successful coping, which would lead them to become more confident in challenging academic environments (i.e., self-efficacy), more optimistic and pro-active in dealing with difficult academic tasks (i.e., optimism), more able to set persistent goals and strategic pathways (i.e., hope), and more able to endure, bounce back, or even grow when confronted by difficulties (i.e., resilience).

The associations of PsyCap and academic stress are extensively studied in education. The significant negative correlation between PsyCap and academic stress was found in teenager students (Gautam and Pradhan 2018) and college students (Yang and Yang 2022). Specifically, the four components of PsyCap are all negatively related to academic stress. For instance, resilience is associated with lower academic stress via effective coping and adaptation (Hu et al. 2015). Hope and optimism are both negatively related to academic stress, and students with a high level of hope or optimism would stick to their goals and look forward to future success (Eden et al. 2020; Lisnyj et al. 2022). Students with high self-efficacy believe that they can conquer the obstacles, therefore, they perceive less stress in learning (Crego et al. 2016; Niazov et al. 2021).

PsyCap and achievement emotions may have a strong mutual relationship. The broaden-and-build theory (Fredrickson 2001) proposes that positive emotions can broaden people’s momentary thought-action repertoires, which serves to build their personal resources including PsyCap. Malinowski and Lim (2015) built a model linking positive affect and the four components of PsyCap. Empirically, Carmona-Halty et al. (2019) found that students’ study-related positive emotions would influence academic performance through the mediating role of PsyCap. Studies also found that PsyCap positively predicts positive emotions and negatively predicts negative emotions (Avey et al. 2008, Da et al. 2021, King et al. 2020). For instance, Kang and Wu (2021) found that school PsyCap can positively predict positive achievement emotions such as enjoyment, hope, and pride. Wang et al. (2021) surveyed 769 Chinese college students and found a negative correlation between PsyCap and negative emotions, and this relationship has been re-examined in several studies (Chevalier et al. 2022; Sahai et al. 2021; Yiwen and Hahn 2021).

Based on the literature, we propose the following hypothesis:

**Hypothesis** **3.** *Psychological capital plays a mediating role in the relationship between creative coping and academic stress as well as in the relationship between creative coping and achievement emotions.*

## 2. Materials and Methods

The current study employed a cross-sectional survey method to collect statistical data. Details are presented below. The study was approved by the University Committee on Human Research Protection of the authors’ institution (HR1-0081-2021).

### 2.1. Participants

Participants were recruited by posters distributed on WeChat, Weibo, online forums, and other platforms from December 2021 to January 2022. Participants who voluntarily joined in the study can scan the QR code in the poster or contact the researcher to complete the survey. Each participant was offered 5RMB after the survey. The initial sample included 1154 Chinese college students; 374 responses were excluded for the following reasons: (1) under the age of 18; (2) failed the attention check; (3) the total response time was unusually short; or (4) filling out the survey more than once using the same account. The effective recovery rate of the questionnaires was 67.6%. The final sample involved 780 college students (57.3% female, Mean age = 21.36 years, SD = 2.00). There were 80 freshmen, 206 sophomores, 205 juniors, 96 seniors, and 153 graduate students. Grade information was missing for the other 40 participants. According to the MedPower software (Kenny 2017), the sample size was considered enough to detect the indirect effects (α set as .05 and the power was virtually 1).

### 2.2. Materials

**Creative coping.** The 10-item simplified version (CCS-10) of the Creative Coping Scale-19 (CCS-19) was used to measure participants’ creative coping (Corry et al. 2013). Items were assessed on a 7-point Likert scale (1 = *disagree strongly* to 7 = *agree strongly*), e.g., “Creativity helps me express my thoughts and feelings”. The Chinese version was back-translated by Psychology and English major graduate students. In this study, the Cronbach’s α of CCS-10 was .90.

**Psychological capital.** The Positive Psychological Capital Questionnaire (PPQ) (Zhang et al. 2010) was utilized. The 26-item scale involved four dimensions: *self-efficacy* (7 items), *resilience* (7 items), *optimism* (6 items), and *hope* (6 items). All items were assessed on a 7-point Likert scale (1 = *complete inconformity* to 7 = *complete conformity*”, e.g., “I feel confident about my ability”. The Cronbach’s α coefficients of the scale in this study was .93.

**Academic stress.** The Learning Stress Inventory for College Students (Tian and Deng 2007) was used to measure participants’ academic stress. The inventory contained 42 items covering seven principal components, namely, future and prospect worry, academic competition pressure, learning efficacy pressure, academic atmosphere pressure, schoolwork burden pressure, learning condition stress, and family expectation pressure. Items were assessed on a 5-point Likert scale (1 = *No such feeling* to 5 = *feeling strongly*). Sample items included “The learning atmosphere in school makes me feel stressful” and “I’m afraid of exams”. The Cronbach’s α coefficients of the inventory in this study was .96.

**Achievement emotions.** Participants’ achievement emotions were measured using the short version of the Achievement Emotions Questionnaire (AEQ-S) (Bieleke et al. 2021; Pekrun et al. 2011). The Chinese version used in this study was back-translated by Psychology and English major graduate students. The 96-item AEQ-S contained class-related emotions, learning-related emotions, and test-related emotions, 32 items each condition. In this study, the items of learning-related emotions were used, measuring three positive emotions (*enjoyment, hope, pride*) and five negative emotions (*anger, anxiety, shame, hopeless,* and *boredom*). Sample items included “I enjoy the challenge of learning the material” and “Studying makes me irritated”. The participants responded to the statements on 5-point Likert scale ranging from 1 (*strongly disagree*) to 5 (*strongly agree*). The Cronbach’s α coefficients of the scale in this study was .88.

### 2.3. Data Analysis

Descriptive statistics, correlation analysis, and regression analysis were conducted using jamovi, a R-based software alternative to SPSS (The jamovi project 2021), to test Hypothesis 1 and Hypothesis 2. Then, since PsyCap is a higher-order variable with four factors, we used Mplus version 8.3 to run structural equation models (SEM) to test Hypothesis 3.

## 3. Results

### 3.1. Descriptive and Correlation Analyses

Descriptive results for the variables, gender differences, and grade differences of the variables can be seen in Table 1. According to the results, skewness and kurtosis were acceptable for all study variables. Independent *t* tests showed that there was a significant gender difference in creative coping (*t* = 4.04, *p* < .001), PsyCap (*t* = 5.26, *p* < .001), and positive achievement emotions (*t* = 2.12, *p* < .05). One-way ANOVA tests showed that there was a significant grade difference in creative coping (*F* = 3.56, *p* < .01), academic stress (*F* = 4.71, *p* < .001), PsyCap (*F* = 4.05, *p* < .001), positive achievement emotions (*F* = 2.16, *p* < .05), and negative achievement emotions (*F* = 3.33, *p* < .01). Table 2 shows the means and standard deviations of examined variables for different grades. According to the results, gender and grade were set as control variables in the subsequent analysis.

Then, correlations of creative coping, PsyCap (the total score and the four factors), academic stress, and achievement emotions (the positive and negative emotions) are shown in Table 3. The results showed that creative coping was positively correlated with positive achievement emotions (*r* = .55, *p* < .001) and negatively correlated with negative achievement emotions (*r* = .08, *p* < .05). Specifically, creative coping was positively associated with enjoyment (*r* = .51, *p* < .01), hope (*r* = .47, *p* < .01), and pride (*r* = .49, *p* < .01), and negatively associations with shame (*r* = −.09, *p* < .05), hopeless (*r* = −.10, *p* < .01), and boredom (*r* = −.11, *p* < .01). Moreover, creative coping was positively associated with PsyCap (*r* = .54, *p* < .001) as well as all the four factors of PsyCap. However, the correlation between creative coping and academic stress was not significant. Hypothesis 1 was partially supported.

### 3.2. Linear Regression Analysis

As the correlation between creative coping and academic stress was not significant, linear regression analysis was conducted only to examine whether creative coping significantly predict achievement emotions. The results are demonstrated in Table 4. Creative coping can positively predict positive achievement emotions (*β* = .36, *t* = 18.17, *p* < .001) and negatively predict negative achievement emotions (*β* = −.07, *t* = −2.15, *p* < .05). Specifically, creative coping can positively predict enjoyment (*β* = .14, *t* = 16.23, *p* < .001), hope (*β* = .15, *t* = 14.79, *p* < .001), and pride (*β* = .14, *t* = 15.86, *p* < .001), and negatively predict anger (*β* = −.33, *t* = −2.01, *p* < .05), shame (*β* = −.04, *t* = −2.47, *p* < .05), hopeless (*β* = −.05, *t* = −2.96, *p* < .01), and boredom (*β* = −.05, *t* = −3.13, *p* < .01). Since the correlation between creative coping and academic stress was not significant, hypothesis 2 was partially supported.

### 3.3. Measurement Models of Research Variables

**Figure 1:**
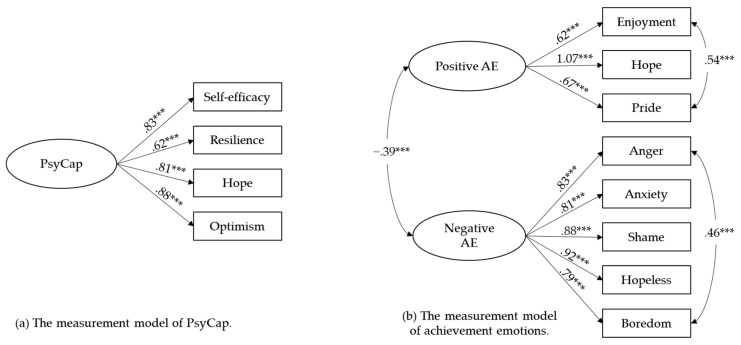
Measurement models of PsyCap and achievement emotions. *Note:* Standardized coefficients are reported. AE = Achievement emotions. Measurement errors and factor loadings are omitted for clarity. *** *p* < .001.

The measurement models of PsyCap and achievement emotions were established before running the whole SEM model (see Figure 1). The model goodness-of-fit after modification are as follows: (a) Chi-Square/df = 2.24; RMSEA = .04; CFI = .999; TLI = .995; SRMR = .006; (b) Chi-Square/df = 5.93; RMSEA = .08; CFI = .98; TLI = .97; SRMR = .04.

### 3.4. The SEM Model

**Figure 2:**
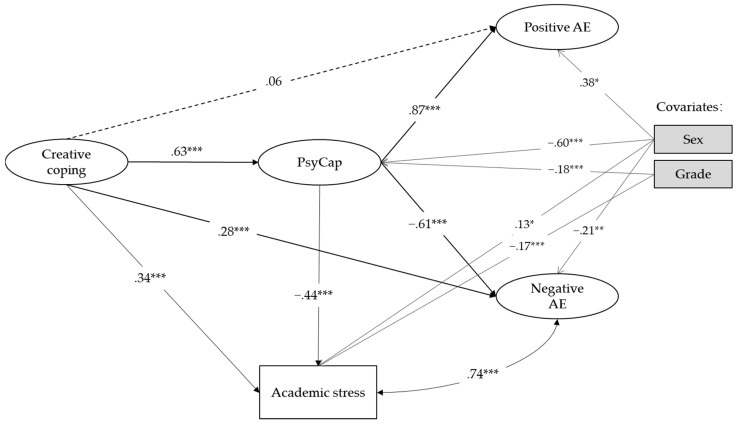
Results of the SEM model. *Note:* N = 780. Standardized parameter estimates are shown. AE = Achievement emotions. Sex and grade were set as control variables. * *p* < .05, ** *p* < .01, *** *p* < .001. The dashed line means insignificant.

We ran an SEM model using Mplus to test the role of PsyCap in the relationship between creative coping and students’ academic stress as well as in the relationship between creative coping and achievement emotions, controlling for sex and grade. We set bootstrap = 5000, and sex and grade as covariates. Figure 2 shows the model (RMSEA = .07, CFI = .92, TLI = .90, SRMR = .07, χ^2^/df = 5.12). The model indirect effect of creative coping on academic stress was significant (estimate = −.28, 95% CI: −.37, −.20), indicating that PsyCap may play a mediating role in the relationship between creative coping and academic stress with a suppression effect (Shrout and Bolger 2002). The indirect effect of creative coping on positive achievement emotions was .54 (*p* < .001, 95% CI: .47, .62), and the indirect effect of creative coping on negative achievement emotions was −.38 (*p* < .001, 95% CI: −.48, −.30). The results showed that PsyCap played a complete mediating role between creative coping and positive achievement emotions and a partial mediating role between creative coping and negative achievement emotions. Hypothesis 3 was supported.

## 4. Discussion

The aim of the study was to test the associations between creative coping and students’ academic stress and achievement emotions and explore the mechanism underpinning the associations. Statistical results indicated that creative coping positively predicted positive achievement emotions and negatively predicted negative achievement emotions via PsyCap. Moreover, PsyCap may play a suppression effect in the relationship between creative coping and students’ academic stress.

The results of the relationship between coping and PsyCap, as well as the relationship between PsyCap and emotions, were largely consistent with the findings of previous studies (e.g., Carmona-Halty et al. 2021; Ding et al. 2015; Yiwen and Hahn 2021; Zhou et al. 2017). As a relatively novel concept, creative coping is considered as one kind of positive coping strategy based on the theory of transformative coping (Corry et al. 2013), which holds the view that creativity can lead to the transformation of negative emotions into positive ones. Although our study did not provide evidence on the change of emotional valence, it supported the claim that creative coping may generate positive effects in enhancing positive achievement emotions, reducing negative achievement emotions, and promoting PsyCap.

It is surprising to find that creative coping did not show significant correlation with academic stress. There is inconsistent evidence on the relationship between coping strategies and academic stress. Some studies have found a significant negative correlation between coping strategies and academic stress (Kuo et al. 2018; Metzger et al. 2017), and some literature shows no significant relationship between coping and academic stress (Hukom and Madrigal 2020; Khan et al. 2014). The inconsistency exists in the relationship between creativity and academic stress as well. Jeong and Park (2004) found that students with higher everyday creativity show lower stress, whereas An et al. (2012) found that students who are novelty-seeking tend to have a higher level of stress. Our study results showed that creative coping was not significantly associated with academic stress in the correlation, but the indirect effect of creative coping on academic stress was significant in the SEM model via the suppression effect of PsyCap as the mediator. These consistencies may be due to the complexity and wide range of academic stress that students experience and further indicate the complicated relationship between creative coping and academic stress. When it comes to the suppression effect of PsyCap, a positive psychological resource, PsyCap was negatively correlated to academic stress, which was consistent with previous studies (Javaheri 2017). Creative coping, as a positive coping method, would build students’ confidence, resilience, optimism, and hope for success in the future, indicating that PsyCap would be enhanced. Through improving PsyCap, creative coping may indirectly reduce students’ self-reported academic stress. Therefore, the association between creative coping and academic stress may be suppressed by PsyCap, and a similar suppression effect can also be seen in other studies (Lv et al. 2021; Mo et al. 2014). Another surprising result was that through applying the SEM Model, the model indirect effect of creative coping on academic stress was significant controlling for gender and grade. Previous studies have also found a positive and significant correlation between creative coping and perceived academic stress (Sohail and Zafar 2022). This result indicates that students need to indulge in more creative endeavors to cope with academic stress.

The current study offers theoretical contributions and educational implications. First, creative coping has been under-researched and this study provides preliminary evidence of its positive relationships with students’ achievement emotions and its potential influence on academic stress via PsyCap. More thorough studies and experiments can be conducted in order to explore the role of creative coping in learning and problem-solving, particularly compared with other coping methods such as emotion- or task-focused coping. Second, it may be useful to design an intervention program to develop students’ capacity of creative coping in the context of academic stress, and it could be part of the existing student counseling programs of developing PsyCap. Third, teachers, school counselors, or educational practitioners may need to develop a supportive, non-judging learning environment for students to feel that their creative coping strategies can be acknowledged and potentially carried out. Moreover, teachers may need to develop a stronger awareness of facilitating students to cope with stressful leaning conditions using various approaches, particularly novel and creative strategies that would raise students’ positive academic emotions and align with students’ personal preferences. For example, teachers can invite their students to experiment with brainstorming a list of creative things to do before examinations and then pick a thing that students usually do not do and execute it. Teachers can encourage students to try creative ways to express their emotions associated with learning, like drawing pictures, taking photos, writing poems, or playing dramas. Last but not least, teachers of different subjects can combine the training of students’ creativity with a specific subject (Pang 2022), so that they may come up with more creative coping strategies when they learn different subjects. 

The current study had several limitations that could be addressed in future research. In terms of methodology, the sample size was small, although this study was capable of detecting medium effects based on power analysis results. From a measurement perspective, we only used self-reported questionnaires to collect data, and this could prevent us from conducting a more nuanced analysis of the relationships of creative coping, academic stress, and achievement emotions. Moreover, the nature of the cross-sectional design was limited in revealing casual effects of creative coping on the dependent variables. Finally, only the unidirectional prediction of creative coping on the learning variable were tested, but we cannot rule out the dynamic reciprocal relations between them. Future research may want to adopt experimental designs to investigate the casual relationships between creative coping and learning variables, and interestingly, a cross-lagged method to test the relationships over time. Task-based assessments of creative coping can be used to reduce common method bias. It might also be of interest to test the effect of creative coping on students’ achievement and success, not just the academic stress, from a positive psychology perspective. We believe that the topic of creative coping and learning calls for more rigorous academic efforts.

## Figures and Tables

**Table 1:** Descriptive statistics for study variables and their gender and grade differences.

	Range	*M*	*SD*	Skewness	Kurtosis	Gender Difference (t)	Grade Difference (F)
**Creative coping**	1–7	5.53	.87	−.81	1.41	4.04 ***	3.56 **
**Academic stress**	1–5	3.35	.76	−.61	.03	−1.30	4.71 ***
**PsyCap**	1–7	5.00	.83	−.41	.80	5.26 ***	4.05 ***
Self-efficacy	1–7	5.17	1.02	−.56	.37	5.34 ***	6.40 ***
Resilience	1–7	4.14	1.12	.26	.05	4.21 ***	2.50 *
Hope	1–7	5.30	.85	−.38	.54	2.82	1.75
Optimism	1–7	5.40	1.02	−.81	1.00	4.84 ***	2.73 *
**Positive AE**	1–5	3.92	.57	−.56	1.00	2.12 *	2.16 *
**Negative AE**	1–5	2.90	.84	.00	−.72	.21	3.33 **

Note: * *p* < .05, ** *p* < .01, *** *p* < .001; AE: Achievement emotion.

**Table 2:** Results of study variables for different grades.

Grade	N	Creative Coping (M ± SD)	Academic Stress (M ± SD)	PsyCap (M ± SD)	Positive AE (M ± SD)	Negative AE (M ± SD)
Freshman	80	5.49 ± .98	3.37 ± .89	5.06 ± .83	3.94 ± .59	2.86 ± .93
Sophomore	206	5.73 ± .82	3.55 ± .76	5.13 ± .72	3.99 ± .53	3.11 ± .88
Junior	205	5.62 ± .81	3.35 ± .78	5.08 ± .89	3.96 ± .63	2.85 ± .91
Senior	96	5.42 ± .87	3.29 ± .70	4.96 ± .89	3.85 ± .59	2.83 ± .75
1st year postgraduate	82	5.37 ± .78	3.13 ± .61	4.99 ± .82	3.86 ± .53	2.71 ± .67
2nd year postgraduate	51	5.39 ± .91	3.37 ± .71	4.64 ± .90	3.90 ± .55	2.87 ± .82
3rd year postgraduate	20	5.22 ± .98	3.14 ± .58	4.62 ± .58	3.73 ± .33	2.83 ± .66

Note: AE: Achievement emotion.

**Table 3:** Correlation analysis of the variables.

	1	2	3	4	5	6	7	8	9	10	11	12	13	14	15	16
**1. Creative coping**	—															
**2. Academic stress**	.06	—														
**3. PsyCap**	.54 ***	−.30 ***	—													
4. Self-efficacy	.55 **	−.13 **	.87 ***													
5. Resilience	.28 **	−.53 **	.76 ***	.52 **	—											
6. Hope	.45 **	−.16 **	.82 ***	.66 **	.42 **	—										
7. Optimism	.54 **	−.14 **	.89 ***	.73 **	.53 **	.72 **	—									
**8. Positive AE**	.55 ***	−.13 ***	.75 ***	.67 ***	.44 ***	.68 ***	.73 ***	—								
9. Enjoyment	.51 **	−.08 *	.60 ***	.54 **	.33 **	.57 **	.58 **	.88 ***	—							
10. Hope	.47 **	−.20 **	.75 ***	.68 **	.50 **	.63 **	.72 **	.89 ***	.66 **	—						
11. Pride	.49 **	−.08 *	.65 ***	.58 **	.33 **	.64 *	.66 **	.91 ***	.72 **	.71 **	—					
**12. Negative AE**	−.08 *	.71 ***	−.44 ***	−.20 ***	−.59 ***	−.37 ***	−.30 ***	−.32 ***	−.24 ***	−.37 ***	−.23 ***	—				
13. Anger	−.07	.57 **	−.37 ***	−.14 **	−.50 **	−.35 **	−.25 **	−.31 ***	−.27 **	−.34 **	−.24 **	.91 ***	—			
14. Anxiety	.06	.69 **	−.27 ***	−.08 *	−.49 **	−.18 **	−.14 **	−.09 *	−.03	−.16 **	−.04	.82 ***	.65 **	—		
15. Shame	−.09 *	.69 **	−.43 ***	−.23 **	−.56 **	−.33 **	−.29 **	−.29 ***	−.19 **	−.36 **	−.21 **	.89 ***	.73 **	.72 **	—	
16. Hopeless	−.10 **	.68 **	−.47 ***	−.23 **	−.58 **	−.39 **	−.35 **	−.33 ***	−.23 **	−.40 **	−.25 **	.92 ***	.78 **	.72 **	.80 **	—
17. Boredom	−.11 **	.54 **	−.39 ***	−.18 **	−.49 **	−.37 **	−.28 **	−.34	−.29 **	−.36 **	−.28 **	.88 ***	.82 **	.59 **	.70 **	.74 **

Note: * *p* < .05. ** *p* < .01. *** *p* < .001; AE = Achievement Emotions.

**Table 4:** Linear regression analysis results of creative coping, academic stress, and achievement emotions.

Dependent Variable	*R*	*β*	*t*
**Positive AE**	.55	.36	18.17 ***
Enjoyment	.50	.14	16.23 ***
Hope	.47	.15	14.79 ***
Pride	.49	.14	15.86 ***
**Negative AE**	.08	−.07	−2.15 *
Anger	.07	−.33	−2.01 *
Anxiety	.06	.02	1.56
Shame	.09	−.04	−2.47 *
Hopeless	.11	−.05	−2.96 **
Boredom	.11	−.05	−3.13 **

Note: * *p* < .05. ** *p* < .01. *** *p* < .001; Independent variable: Creative coping; AE = Achievement emotion.

## Data Availability

The data are currently not publicly available due to participant privacy.

## References

[B1-jintelligence-10-00126] Alfonso-Benlliure Vicente, Melendez Juan Carlos (2022). Creativity as a “vaccine” for depressed mood: Coping and divergent thinking in young adults. Annals of Psychology.

[B2-jintelligence-10-00126] Alzahrani Ahmed M., Hakami Ahmed, AlHadi Ahmad, Batais Mohammed A., Alrasheed Abdullah A., Almigbal Turky H. (2020). The interplay between mindfulness, depression, stress and academic performance in medical students: A Saudi perspective. PLoS ONE.

[B3-jintelligence-10-00126] An Hoyoung, Chung Seockhoon, Park Jangho, Kim Seong-Yoon, Kim Kyung Mo, Kim Ki-Soo (2012). Novelty-seeking and avoidant coping strategies are associated with academic stress in Korean medical students. Psychiatry Research.

[B4-jintelligence-10-00126] Arsenio William F., Loria Samantha (2014). Coping with negative emotions: Connections with adolescents’ academic performance and stress. The Journal of Genetic Psychology.

[B5-jintelligence-10-00126] Avey James B., Wernsing Tara S., Luthans Fred (2008). Can positive employees help positive organizational change? Impact of psychological capital and emotions on relevant attitudes and behaviors. The Journal of Applied Behavioral Science.

[B6-jintelligence-10-00126] Bieleke Maik, Gogol Katarzyna, Goetz Thomas, Daniels Lia, Pekrun Reinhard (2021). The AEQ-S: A short version of the Achievement Emotions Questionnaire. Contemporary Educational Psychology.

[B7-jintelligence-10-00126] Cai Wenjing, Lysova Evgenia I., Bossink Bart A. G., Khapova Svetlana N., Wang Weidong (2019). Psychological capital and self-reported employee creativity: The moderating role of supervisor support and job characteristics. Creativity and Innovation Management.

[B8-jintelligence-10-00126] Carmona-Halty Marcos, Salanova Marisa, Llorens Susana, Schaufeli Wilmar B. (2019). How psychological capital mediates between study–related positive emotions and academic performance. Journal of Happiness Studies.

[B9-jintelligence-10-00126] Carmona-Halty Marcos, Salanova Marisa, Llorens Susana, Schaufeli Wilmar B. (2021). Linking positive emotions and academic performance: The mediated role of academic psychological capital and academic engagement. Current Psychology.

[B10-jintelligence-10-00126] Carver Charles S., Scheier Michael F., Weintraub Jagdish Kumari (1989). Assessing coping strategies: A theoretically based approach. Journal of Personality and Social Psychology.

[B11-jintelligence-10-00126] Chen Xinjie, Padilla Amado M. (2019). Emotions and creativity as predictors of resilience among L3 learners in the Chinese educational context. Current Psychology.

[B12-jintelligence-10-00126] Cherkil Sandhya, Gardens Seby J., Soman Deepak Kuttikatt (2013). Coping styles and its association with sources of stress in undergraduate medical students. Indian Journal of Psychological Medicine.

[B13-jintelligence-10-00126] Cherry Megan L., Wilcox Melanie M. (2020). Decreasing perceived and academic stress through emotion regulation and nonjudging with trauma-exposed college students. International Journal of Stress Management.

[B14-jintelligence-10-00126] Chevalier Séverine, Calmé Isabelle, Coillot Hélène, Rudulier Karine Le, Fouquereau Evelyne (2022). How can students’ entrepreneurial intention be increased? The role of psychological capital, perceived learning from an entrepreneurship education program, emotions and their relationships. Europe’s Journal of Psychology.

[B15-jintelligence-10-00126] Conner Tamlin S., DeYoung Colin G., Silvia Paul J. (2018). Everyday creative activity as a path to flourishing. The Journal of Positive Psychology.

[B16-jintelligence-10-00126] Corry Dagmar A. S., Lewis Christopher Alan, Mallett John (2014). Harnessing the mental health benefits of the creativity-spirituality construct: Introducing the Theory of Transformative Coping. Journal of Spirituality in Mental Health.

[B17-jintelligence-10-00126] Corry Dagmar Anna Susanne, Mallett John, Lewis Christopher Alan, Abdel-Khalek Ahmed M (2013). The creativity-spirituality construct and its role in transformative coping. Mental Health, Religion & Culture.

[B18-jintelligence-10-00126] Crego Antonio, Carrillo-Diaz María, Armfield Jason M., Romero Martín (2016). Stress and academic performance in Dental students: The role of coping strategies and examination-related self-efficacy. Journal of Dental Education.

[B19-jintelligence-10-00126] Da Shu, Zhu Ze, Cen Hongyu, Gong Xianmin, Siu Oi Ling, Zhang Xichao (2021). Psychological Capital, positive affect, and organizational outcomes: A three-wave cross-lagged study. Journal of Pacific Rim psychology.

[B20-jintelligence-10-00126] Darabi Mitra, Macaskill Ann, Reidy Lisa (2017). Stress among UK academics: Identifying who copes best. Journal of Further and Higher Education.

[B21-jintelligence-10-00126] De Fazio Caterina, Dennis Tiffany, English Rachel, Sattelmeier Brent (2019). Creative Coping Strategies: Reducing Academic Stress in College Students.

[B22-jintelligence-10-00126] Denovan Andrew, Macaskill Ann (2017). Stress, resilience and leisure coping among university students: Applying the broaden-and-build theory. Leisure Studies.

[B23-jintelligence-10-00126] Ding Xiaoqian, Tang Yi-Yuan, Tang Rongxiang, Posner Michael I. (2014). Improving creativity performance by short-term meditation. Behavioral and Brain Functions.

[B24-jintelligence-10-00126] Ding Yongqing, Yang Yanjie, Yang Xiuxian, Zhang Tiehui, Qiu Xiaohui, He Xin, Wang Wenbo, Wang Lin, Sui Hong (2015). The mediating role of coping style in the relationship between psychological capital and burnout among Chinese nurses. PLoS ONE.

[B25-jintelligence-10-00126] Eden Allison L., Johnson Benjamin K., Reinecke Leonard, Grady Sara M. (2020). Media for coping during COVID-19 social distancing: Stress, anxiety, and psychological well-being. Frontiers in Psychology.

[B26-jintelligence-10-00126] Falat Marek (2000). Creativity as a predictor of “good” coping?. Studia Psychologica.

[B27-jintelligence-10-00126] Folkman Susan, Lazarus Richard S. (1980). An analysis of coping in a middle-aged community sample. Journal of Health and Social Behavior.

[B28-jintelligence-10-00126] Folkman Susan (1984). Personal control and stress and coping processes: A theoretical analysis. Journal of Personality and Social Psychology.

[B29-jintelligence-10-00126] Folkman Susan (2010). Stress, coping, and hope. Psycho-Oncology.

[B30-jintelligence-10-00126] Fredrickson Barbara L. (2001). The role of positive emotions in positive psychology: The Broaden-and-Build Theory of positive emotions. The American Psychologist.

[B31-jintelligence-10-00126] Gautam Priyanka, Pradhan Madhurima (2018). Psychological capital as moderator of stress and achievement. Indian Journal of Positive Psychology.

[B32-jintelligence-10-00126] Gocłowska Małgorzata A., Ritter Simone M., Elliot Andrew J., Baas Matthijs (2019). Novelty seeking is linked to openness and extraversion, and can lead to greater creative performance. Journal of Personality.

[B33-jintelligence-10-00126] Harley Jason M., Pekrun Reinhard, Taxer Jamie L., Gross James J. (2019). Emotion regulation in achievement situations: An integrated model. Educational Psychologist.

[B34-jintelligence-10-00126] Hu Tianqiang, Zhang Dajun, Wang Jinliang (2015). A meta-analysis of the trait resilience and mental health. Personality and Individual Differences.

[B35-jintelligence-10-00126] Huang Lei, Luthans Fred (2015). Toward better understanding of the learning goal orientation–creativity relationship: The role of positive psychological capital. Applied Psychology.

[B36-jintelligence-10-00126] Hukom Kenia, Madrigal Dennis (2020). Assessing the correlation between demographics, academic stress, and coping strategies of filipino high school students with single-parents. Philippine Social Science Journal.

[B37-jintelligence-10-00126] Javaheri Abbas (2017). Psychological Capital: An Internal Resource for Counseling Students Coping with Academic and Clinical Stress.

[B38-jintelligence-10-00126] Jeong Eun I., Park Yonghan (2004). The relationships between everyday creativity, stress and stress coping strategies of college students. Korean Journal of Educational Research.

[B39-jintelligence-10-00126] Kang Xia, Wu Yajun (2021). Investigating the linkage between school psychological capital and achievement emotions in secondary school mathematics. The Asia-Pacific Education Researcher.

[B40-jintelligence-10-00126] Kausar Rukhsana (2010). Perceived stress, academic workloads and use of coping strategies by university students. Journal of Behavioural Sciences.

[B41-jintelligence-10-00126] Kenny David A. (2017). MedPower: An Interactive Tool for the Estimation of Power in Tests of Mediation [Computer Software]. https://davidakenny.shinyapps.io/MedPower/.

[B42-jintelligence-10-00126] Khan Aqeel, Ahmad Roslee, Hamdan Abdul Rahim, Mustaffa Mohamed Sharif, Tahir Lokman Mohd (2014). Does psychological strengths and subjective well-being predicting parental involvement and problem solving among Malaysian and Indian students?. SpringerPlus.

[B43-jintelligence-10-00126] Kimport Elizabeth R., Robbins Steven J. (2012). Efficacy of creative clay work for reducing negative mood: A randomized controlled trial. Art Therapy.

[B44-jintelligence-10-00126] King Ronnel B., Pitliya Riddhi J., Datu Jesus A. (2020). Psychological capital drives optimal engagement via positive emotions in work and school contexts. Asian Journal of Social Psychology.

[B45-jintelligence-10-00126] Kuo Ben C. H., Soucie Kendall M., Huang Siqi, Laith Refa (2018). The mediating role of cultural coping behaviours on the relationships between academic stress and positive psychosocial well-being outcomes. International Journal of Psychology.

[B46-jintelligence-10-00126] Lazarus Richard S., Folkman Susan (1984). Stress, Appraisal, and Coping.

[B47-jintelligence-10-00126] Leroy Véronique, Grégoire Jacques, Magen Eran, Gross James J., Mikolajczak Moïra (2012). Resisting the sirens of temptation while studying: Using reappraisal to increase focus, enthusiasm, and performance. Learning and Individual Differences.

[B48-jintelligence-10-00126] Li Fuli, Chen Tingting, Lai Xin (2018). How does a reward for creativity program benefit or frustrate employee creative performance? The perspective of Transactional Model of Stress and Coping. Group & Organization Management.

[B49-jintelligence-10-00126] Lisnyj Konrad, Pearl David L., McWhirter Jennifer E., Papadopoulos Andrew (2022). Examining the influence of human and psychological capital variables on post-secondary students’ academic stress. Studies in Higher Education.

[B50-jintelligence-10-00126] Liu Xianwei, Zou Yang, Ma Yonghong, Gao Wei (2020). What affects PhD student creativity in China? A case study from the Joint Training Pilot Project. Higher Education.

[B51-jintelligence-10-00126] Luthans Fred, Avolio Bruce J., Youssef Carolyn M. (2006). Psychological Capital: Developing the Human Competitive Edge.

[B52-jintelligence-10-00126] Luthans Fred, Avolio Bruce J., Avey James B., Norman Steven M. (2007). Positive psychological capital: Measurement and relationship with performance and satisfaction. Personnel Psychology.

[B53-jintelligence-10-00126] Luthans Fred, Luthans Kyle W., Luthans Brett C. (2004). Positive psychological capital: Beyond human and social capital. Business Horizons.

[B54-jintelligence-10-00126] Lv Miao, Tan Xuyun, Xing Cai, Zheng Jiaren, Han Sixuan (2021). How family-work conflict influences post-traumatic growth among medical workers: A moderated mediation model. Frontiers in Psychology.

[B55-jintelligence-10-00126] Malinowski Peter, Lim Hui Jia (2015). Mindfulness at work: Positive affect, hope, and optimism mediate the relationship between dispositional mindfulness, work engagement, and well-being. Mindfulness.

[B56-jintelligence-10-00126] Metzger Isha W., Blevins Claire, Calhoun Casey D., Ritchwood Tiarney D., Gilmore Amanda K., Stewart Regan, Bountress Kaitlin E. (2017). An examination of the impact of maladaptive coping on the association between stressor type and alcohol use in college. Journal of American College Health.

[B57-jintelligence-10-00126] Mirsamiei Marzieh, Atashpour Hamid, Aghaei Asghar (2021). Effect of achievement emotion regulation training package on negative emotions and learning strategies among female high school students. Journal of Research & Health.

[B58-jintelligence-10-00126] Mo Ruo, Leung Louis, Hao Yingqi, Wu Xuan, Xi Rui, Zhang Shu (2014). Examining the mediating roles of microblog use in the relationships between narcissism, social anxiety, and social capital. International Journal of Cyber Behavior, Psychology, and Learning.

[B59-jintelligence-10-00126] Neveu Dorine, Doron Julie, Visier Laurent, Boiché Julie, Trouillet Raphaël, Dujols Pierre, Ninot Gregory (2012). Students perceived stress in academic programs: Consequences for its management. Revue Depidemiologie et de Sante Publique.

[B60-jintelligence-10-00126] Niazov Zehava, Hen Meirav, Ferrari Joseph R. (2021). Online and academic procrastination in students with learning disabilities: The impact of academic stress and self-efficacy. Psychological Reports.

[B61-jintelligence-10-00126] Norma Contini de González (2016). Creativity as a coping resource in daily life. La Creatividad Como Recurso de Afrontamiento en la Vida Cotidiana.

[B62-jintelligence-10-00126] Pang Weiguo (2022). Psychological views on cultivating students’ creativity: Goals, principles, and strategies. Journal of East China Normal University (Educational Sciences).

[B63-jintelligence-10-00126] Park Se-Hyuk, Kim Youngshim (2018). Ways of coping with excessive academic stress among Korean adolescents during leisure time. International Journal of Qualitative Studies on Health and Well-Being.

[B64-jintelligence-10-00126] Pavlova Elizaveta M., Kornilova Tatyana V. (2013). Creativity and tolerance for uncertainty predict the engagement of emotional intelligence in personal decision making. Psychology in Russia: State of the Art.

[B65-jintelligence-10-00126] Pekrun Reinhard (2006). The control-value theory of achievement emotions: Assumptions, corollaries, and implications for educational research and practice. Educational Psychology Review.

[B66-jintelligence-10-00126] Pekrun Reinhard, Lichtenfeld Stephanie, Marsh Herbert W., Murayama Kou, Goetz Thomas (2017). Achievement emotions and academic performance: Longitudinal models of reciprocal effects. Child Development.

[B67-jintelligence-10-00126] Pekrun Reinhard, Goetz Thomas, Frenzel Anne C., Barchfeld Petra, Perry Raymond P. (2011). Measuring emotions in students’ learning and performance: The Achievement Emotions Questionnaire (AEQ). Contemporary Educational Psychology.

[B68-jintelligence-10-00126] Putwain David W., Daly Anthony L., Chamberlain Suzanne, Sadreddini Shireen (2016). “Sink or swim”: Buoyancy and coping in the cognitive test anxiety—Academic performance relationship. Educational Psychology.

[B69-jintelligence-10-00126] Rabenu Edna, Yaniv Eyal, Elizur Dov (2016). The relationship between Psychological Capital, coping with stress, well-being, and performance. Current Psychology.

[B70-jintelligence-10-00126] Sahai Shikha, Ciby Mariam Anil, Dominic Elizabeth (2021). Workplace isolation amongst home-based teleworkers: Can psychological capital make a difference?. Human Systems Management.

[B71-jintelligence-10-00126] Salimzadeh Raheleh, Hall Nathan C., Saroyan Alenoush (2021). Examining academics’ strategies for coping with stress and emotions: A review of research. Frontiers in Education.

[B72-jintelligence-10-00126] Santiago Catherine DeCarlo, Brewer Stephanie K., Fuller Anne K., Torres Stephanie A., Papadakis Jaclyn Lennon, Ros Anna M. (2017). Stress, coping, and mood among Latino adolescents: A daily diary study. Journal of Research on Adolescence.

[B73-jintelligence-10-00126] Shrout Patrick E., Bolger Niall (2002). Mediation in experimental and nonexperimental studies: New procedures and recommendations. Psychological Methods.

[B74-jintelligence-10-00126] Sohail Marva, Zafar Nida (2022). Fear of COVID-19 and stress in university students: Mediating role of cyberchondria and moderating role of creative coping and social supports. Journal of the Pakistan Medical Association.

[B75-jintelligence-10-00126] Stoliker Bryce E., Lafreniere Kathryn D. (2015). The influence of perceived stress, loneliness, and learning burnout on university students’ educational experience. College Student Journal.

[B76-jintelligence-10-00126] Strain Amber Chauncey, D’Mello Sidney K. (2015). Affect regulation during learning: The enhancing effect of cognitive reappraisal. Applied Cognitive Psychology.

[B77-jintelligence-10-00126] Stupnisky Robert H., Perry Raymond P., Renaud Robert D., Hladkyj Steve (2013). Looking beyond grades: Comparing self-esteem and perceived academic control as predictors of first-year college students’ well-being. Learning and Individual Differences.

[B78-jintelligence-10-00126] The jamovi project (2021). jamovi (Version 1.6) [Computer Software]. https://www.jamovi.org.

[B79-jintelligence-10-00126] Thomas Christopher L., Cassady Jerrell C., Heller Monica L. (2017). The influence of emotional intelligence, cognitive test anxiety, and coping strategies on undergraduate academic performance. Learning and Individual Differences.

[B80-jintelligence-10-00126] Tian Lan, Deng Qi (2007). Development of learning stress inventory for college students. Chinese Journal of Behavioral Medicine Science.

[B81-jintelligence-10-00126] Wang Wei, Xu Huiying, Wang Bingmei, Zhu Enzhi (2019). The mediating effects of learning motivation on the association between perceived stress and positive-deactivating academic emotions in nursing students undergoing skills training. Journal of Korean Academy of Nursing.

[B82-jintelligence-10-00126] Wang Wenbo, Mehmood Anam, Li Ping, Yang Zhaonan, Niu Jinbao, Chu Haiyun, Qiao Zhengxue, Qiu Xiaohui, Zhou Jiawei, Yang Yanjie (2021). Perceived stress and smartphone addiction in medical college students: The mediating role of negative emotions and the moderating role of psychological capital. Frontiers in Psychology.

[B83-jintelligence-10-00126] Wu Mingchang, Siswanto Ibnu, Ko Chenju (2017). The influential factors and hierarchical structure of college students’ creative capabilities—An empirical study in Taiwan. Thinking Skills and Creativity.

[B84-jintelligence-10-00126] Yang Yong, Yang Pingzhan (2022). Effect of college students’ academic stress on anxiety under the background of the normalization of COVID-19 pandemic: The mediating and moderating effects of psychological capital. Frontiers in Psychology.

[B85-jintelligence-10-00126] Yeh Yu-Chu, Li Me-Lin (2008). Age, emotion regulation strategies, temperament, creative drama, and preschoolers’ creativity. The Journal of Creative Behavior.

[B86-jintelligence-10-00126] Yiwen Fei, Hahn Juhee (2021). Job insecurity in the COVID-19 pandemic on counterproductive work behavior of millennials: A time-lagged mediated and moderated model. International Journal of Environmental Research and Public Health.

[B87-jintelligence-10-00126] Yousif Mariam A., Arbab Ahmed H., Yousef Bashir A. (2022). Perceived academic stress, causes, and coping strategies among undergraduate pharmacy students During the COVID-19 pandemic. Advances in Medical Education and Practice.

[B88-jintelligence-10-00126] Zhang Jieting, Zheng Yao (2017). How do academic stress and leisure activities influence college students’ emotional well-being? A daily diary investigation. Journal of Adolescence.

[B89-jintelligence-10-00126] Zhang Kuo, Zhang Sai, Dong Yinghong (2010). Positive psychological capital: Measurement and relationship with mental health. Studies of Psychology and Behavior.

[B90-jintelligence-10-00126] Zhang Yunshu, Liu Wenling, Liu Ying, Huang Zhaoming, Liu Qiaoyun (2019). Chinese college students’ optimism and social creativity mediated by creative self-efficacy and hope. Social Behavior and Personality.

[B91-jintelligence-10-00126] Zhou Hongzhen, Peng Juan, Wang D., Kou L., Chen F., Ye M., Deng Y., Yan J., Liao S. (2017). Mediating effect of coping styles on the association between psychological capital and psychological distress among Chinese nurses: A cross-sectional study. Journal of Psychiatric and Mental Health Nursing.

[B92-jintelligence-10-00126] Zyberaj Jetmir (2022). Investigating the relationship between emotion regulation strategies and self-efficacy beliefs among adolescents: Implications for academic achievement. Psychology in the Schools.

